# Development and psychometric evaluation of the Motivation to Use CPAP Scale (MUC-S) using factorial structure and Rasch analysis among patients with obstructive sleep apnea before CPAP treatment is initiated

**DOI:** 10.1007/s11325-020-02143-9

**Published:** 2020-07-23

**Authors:** Anders Broström, M. Ulander, P. Nilsen, Chung-Ying Lin, A. H. Pakpour

**Affiliations:** 1 Department of Nursing, School of Health and Welfare, Jönköping University, Jönköping, Sweden; 2 Department of Clinical Neurophysiology, Linköping University Hospital, S-581 85 Linköping, Sweden; 3 Department of Clinical and Experimental Medicine, Division of Clinical Neurophysiology, Faculty of Health Sciences, Linköping University, Linköping, Sweden; 4 Department of Health, Medicine and Caring Sciences, Division of Public Health, Faculty of Health Sciences, Linköping University, Linköping, Sweden; 5 Department of Rehabilitation Sciences, The Hong Kong Polytechnic University, Hung Hom, Hong Kong; 6 Social Determinants of Health Research Center, Qazvin University of Medical Sciences, Shahid Bahounar BLV, Qazvin, 3419759811 Iran

**Keywords:** Obstructive sleep apnea, Continuous positive airway treatment, Ethos, Validity, Reliability

## Abstract

**Background:**

Continuous positive airway treatment (CPAP) is first-line treatment for obstructive sleep apnea (OSA), but adherence tends to be low. A clinical tool focusing on motivation to use CPAP is missing. The purpose was to develop a brief questionnaire to assess motivation to use CPAP that is psychometrically robust and suitable for use in clinical practice.

**Methods:**

A convenience sample including 193 treatment naive patients with OSA (67% men; mean age = 59.7 years, SD 11.5) from two CPAP clinics was used. Clinical assessments and full night polygraphy were performed. Questionnaires administered before CPAP treatment included the newly developed Motivation to Use CPAP Scale (MUC-S), Minimal Insomnia Symptoms Scale (MISS), Epworth Sleepiness Scale (ESS), and Attitude towards CPAP treatment Inventory (ACTI). The validity and reliability of the MUC-S were investigated using Rasch and exploratory factor analysis models. Measurement invariance, dimensionality and differential item functioning (i.e., across gender groups, excessive daytime sleepiness (ESS), insomnia (MISS) and attitude towards CPAP (ACTI) groups) were assessed.

**Results:**

The results supported a two-factor solution (autonomous motivation, 6 items, factor loadings between 0.61 and 0.85 and controlled motivation, 3 items, factor loadings between 0.79 and 0.88) explaining 60% of the total variance. The internal consistency was good with Cronbach’s alpha of 0.88 and 0.86 for the two factors. No differential item functioning was found. A latent class analysis yielded three profiles of patients with high (*n* = 111), moderate (*n* = 60) and low (*n* = 22) motivation. Patients with high motivation were older, had higher daytime sleepiness scores, more insomnia symptoms and a more positive attitude towards CPAP.

**Conclusions:**

The MUC-S seems to be a valid tool with robust psychometric properties suitable for use at CPAP clinics. Future studies should focus on how motivation changes over time and if MUC-S can predict objective long-term CPAP adherence.

## Introduction

Obstructive sleep apnea (OSA) is a prevalent and successively increasing multifaceted condition influencing the whole life situation of the patient [1, 2]. Continuous positive airway pressure (CPAP) is the preferred treatment alternative [3], especially for moderate and severe OSA, which if optimally used leads to reduced symptoms, improved metabolic control, lowered cardiovascular morbidity, as well as decreased all-cause and cardiovascular mortality [4]. Despite these effects, patients often struggle during treatment initiation causing numerous early dropouts [5, 6]. Long-term CPAP adherence, often defined as > 4 h use on 70% of the nights [7] vary greatly [8] and is generally seen as a substantial clinical problem [9]. A dose-response relationship between CPAP usage and a variety of outcomes seem to exist, and the optimal adherence level differs depending on the outcome in question. Importantly, sleep duration should also be considered, and a usage level as high as possible is desirable [6]. Two recent review studies [10, 11] stated that socio-demographic characteristics (e.g., age, gender, socio economic status), symptoms and disease severity (e.g., daytime sleepiness and AHI), as well as treatment aspects (e.g., initiation procedure, side-effects), apart from symptomatic improvement, generally show limited predictive power for CPAP adherence. On the other hand, psychosocial variables (e.g., attitude, self-efficacy, illness and treatment beliefs or social support) have been found to be vital during the initiation in studies using patient- [12–17], partner- [18, 19] and practitioner-centred perspectives [9, 20] and ought to be thought of as probable predictors for CPAP use [6].

The initiation of CPAP is a complex procedure although it can be simplified and made more effective by a good interaction between patient and practitioner [16, 20]. A recent survey including all CPAP centres in Sweden and Norway showed that practitioners perceived patients’ motivation, attitudes and knowledge to be the main determinants of CPAP adherence, but educating patients about effects, management and treatment adjustments were the most common actions they used to improve adherence [9]. The value of basing CPAP treatment on theory and include psychosocial variables to understand the mechanisms of change and predictors of CPAP adherence has been stressed [10]. A comprehensive and well-supported theory to understand human motivation is the Self-determination theory (SDT) [21]. SDT posits that all behaviours lie along a continuum of relative autonomy, i.e., self-determination, mirroring the degree to which a person supports what he or she is doing. At one end of the self-determination continuum is behaviour that is intrinsically motivated and executed for its natural satisfaction, e.g., for the fun, curiosity or challenge it offers. At the other end is amotivation, which refers to a lack of intent to execute the behaviour. In between intrinsically motivated behaviours and amotivation lie behaviours that are described as extrinsic, suggesting that they are done to obtain certain outcomes contrary to intrinsic behaviours, which are done for their own sake. Four types of extrinsically motivated behaviours are recognized: integrated (i.e., behaviours consistent with a person’s values and needs, done because they signify what the person stands for), identified (i.e., behaviours experienced as beneficial to a person’s development, but not necessarily done with enjoyment), introjected (i.e., behaviours done to avoid negative feelings such as guilt or shame) and externally regulated (i.e., behaviours done to satisfy an external demand or reward contingency). Introjected and external regulations are portrayed as controlled motivation, whereas intrinsic, integrated and identified types of motivation are labelled autonomous motivation. A substantial body of research exists that shows that more autonomously motivated behaviours are more stable, performed with greater care and quality and accompanied by more positive experiences [21]. Despite wide acknowledgment of the significance of motivation to use CPAP and adhere to the treatment, a validated questionnaire to measure and quantify motivation among patients with OSA is missing. By using a validated instrument when initiating CPAP, practitioners can identify patients with low motivation and after exploring causes subsequently improve adherence through dealing with identified reasons. The aim of this study was to develop a brief questionnaire to assess motivation to use CPAP that is psychometrically robust and suitable for use in clinical practice.

## Materials and methods

### Development and description of the Motivation to Use CPAP Scale (MUC-S)

Initially, qualitative studies were identified by three members of the research group (i.e., a physician, a nurse and a behavioural scientist) to receive an in-depth understanding of the patient, partner and practitioner perspective of motivation to use CPAP [14, 16, 18–20, 22, 23]. In the second step, several review studies summarizing factors associated to CPAP adherence (e.g., 8), with specific focus on behavioural aspects (e.g., 10), were studied. In the third step, the three researchers used their clinical experience, knowledge of the SDT and understanding of the reviewed literature to inspire the development of a pool of potential items (i.e., 15 items) aimed to measure different aspects of motivation to use CPAP. In the fourth step, eight new persons joined the group: two physicians (i.e., clinical experts and distinguished CPAP adherence researchers), five nurses (i.e., clinical experts with long experience working with CPAP treatment), as well as one nurse researcher with extensive experience of instrument development. The aim was to discuss and mutually establish content validity (i.e., finding items with a high level of clinical significance). All 15 items were scrutinized based on the group’s clinical- and research-related understanding of the concept and scored either as inappropriate, or as appropriate for measuring motivation to use CPAP, and inclusion in the scale. A consensus discussion generated 12 items that was intended to span different regulatory styles from the SDT [21] describing autonomous motivation, controlled motivation and amotivation to use CPAP treatment. In the fifth step, the instrument was pilot tested on a group of 10 CPAP patients who confirmed layout, content, wording and readability of the 12 items. A 5-point Likert-type scale from strongly agree (5) to strongly disagree (1) was deemed appropriate for each item. The final version of MUC-S is presented in Table 7. The possible range for the scale is 9–45. A higher score indicates greater motivation to use CPAP treatment.

### Design and population

This was a psychometric validation study with a cross-sectional design including 193 consecutive treatment-naive patients with OSA from two CPAP clinics at one university and one county hospital in southern Sweden. Inclusion criteria were objectively verified OSA, receiving CPAP treatment for the first time. The following exclusion criteria were used: terminal disease, ongoing treatment for OSA, severe psychiatric disease, dementia, alcohol/drug abuse or difficulties reading and understanding the Swedish language. All data were collected before CPAP was initiated.

### Data collection

#### Clinical variables

Clinical variables, co-morbidities, blood pressure and body mass index were collected from medical records or face to face during clinical examinations at the CPAP clinics before treatment was initiated. An all-night home-based polygraphy (Embletta, ResMed Sweden AB) including nasal airflow, pulse oximetry, breathing movements and posture, assessed by an experienced sleep specialist, was used to diagnose OSA.

### Questionnaires

#### Insomnia

The main features of insomnia (i.e., difficulties initiating sleep, difficulties maintaining sleep, as well as difficulties with non-restorative sleep) were measured by the validated Minimal insomnia symptoms scale (MISS) [24]. The three items focus on perceived difficulties on each one of the features with scales ranging from no problems (0) to very great problems (4). A total score of 0–3 implies no clinical insomnia, 4–6 subclinical insomnia, 7–9 moderate clinical insomnia and 10–12 severe clinical insomnia.

#### Excessive daytime sleepiness

Excessive daytime sleepiness was measured by the comprehensively validated Epworth sleepiness scale (ESS) [25]. Eight various daily situations are used where patients score (i.e., 0–3) the probability of dozing or falling asleep. The total score of ESS range from 0 to 24 points, with a cut-off of > 10 indicating excessive daytime sleepiness.

#### Global perceived health

The first item regarding present health status from the SF-36 was applied to measure global perceived health [26]. The patients rated their health as (1) excellent, (2) very good, (3) good, (4) fair or (5) poor.

#### Attitudes towards CPAP treatment

The validated Attitudes to CPAP treatment inventory (ACTI) was used to assess attitudes towards CPAP treatment [15]. The five items are rated on a 5-point Likert-type scale and range from strongly agree (1) to strongly disagree (5). A higher total score indicates a more negative attitude towards CPAP treatment.

### Statistical analysis

Descriptive statistics were used to calculate data regarding baseline characteristics. Normally distributed clinical variables on an interval scale were presented with mean ± standard deviations (SD) or in the case of categorical variables as *n* (%).

To perform a satisfactory validation and psychometric testing of a new instrument, such as MUC-S, two testing theories (i.e., classical test theory [CTT] and modern test theory, such as Rasch models) are suggested to be applied concurrently [27–30]. CTT as compared with Rasch uses more basic mathematic methods to assess the psychometric properties of an instrument. On the other hand, Rasch models have the advantages of a rigorous in additive calculation, the lack of which is a major limitation in the CTT. Also, Rasch is sample-free (i.e., the psychometric properties obtained from Rasch are not affected by the sample characteristics), while CTT suffers the weakness of sample-dependency [27, 31]. Both CTT and modern test theory were used in the psychometric validation of MUC-S.

#### Reliability

Cronbach’s alpha and item-total correlation were used to evaluate internal consistency, with values of > 0.7 and > 0.4 implying satisfactory internal consistency. The standard error of measurement was calculated to evaluate measurement errors in the two factors originated from the exploratory factor analysis. A value of < SD/2 suggests a satisfactory measurement error [32].The percentages of missing data for each included item, as well as floor (i.e., the proportion of patients with the worst possible score) and ceiling effects (i.e., the proportion of patients with the best possible score), were calculated. Values > 20% indicate the presence of floor or ceiling effects [33].

#### Validity

An exploratory factor analysis was applied (i.e., a principal axis factoring analysis with two factors rotated using a direct oblimin procedure) to reveal the underlying structure of the scale [34, 35]. Bartlett’s test of sphericity with Kaiser–Meyer–Olkin measure was used for each item, and all items together to indicate sampling adequacy. The Kaiser criteria (eigenvalue > 1.0) was used to determine the number of extracted factors, and factor loadings > 0.4 were judged as significant according to the sample size [35].

Rasch modelling [31] (using the WINSTEPS Rasch Analysis software version 4.01) was applied to evaluate local dependency, item validity, item and person separation reliabilities and item and person separation indices, as well as differential item functioning. Two fit indices were used to measure item validity: information-weighted fit statistic (infit) mean square and outlier-sensitive fit statistic (outfit) mean square, with a suggested range between 0.5 and 1.5 to show satisfactory fit to the model. Values > 0.7 were judged tolerable for item and person reliability. The capability of the items and individuals to divide into two or more distinct groups was assessed by means of item and person indices, with values > 2 being acceptable. Moreover, analysis of differential item functioning was conducted to make sure that subgroups of patients (based on gender, sleepiness (i.e., ESS score > 10 vs ≤ 10) and insomnia (i.e., MISS score > 7 vs ≤ 7) groups) could interpret the MUC-S items equally. A differential item functioning contrast greater than 0.5 logits indicates a noticeable and non-ignorable difference in item interpretation between groups with different characteristics.

#### Latent class analysis

Latent class analyses (performed in Mplus 7.3) were used to define complex individual-centred pattern of associations [36]. The following fit indices were used to select an appropriate model for motivation: the Akaike information criterion, the Bayesian information criterion, the sample-size-adjusted Bayesian information criterion, entropy and the adjusted Lo-Mendell-Rubin’s likelihood ratio test [37]. In line with recommendations, an acceptable model fit was indicated by lower values on Akaike information criterion, Bayesian information criterion and sample-size-adjusted Bayesian information criterion. Also, higher values on entropy suggest better classification. We also used the Lo-Mendell-Rubin’s likelihood ratio test, where a significant result reveals that a K-class model fits the data better than a (K-1)-class model [37].

## Results

### Study population

Patient demographics and clinical data are shown in Table 1. A total of 193 patients (mean age = 59.7 years, SD 11.5, range 20–84) participated, of which 68% were males and 61% were married. The mean AHI was 35.6 (SD 18.7, range 10–94) and 10%, 27% and 63% of the patients suffered from mild, moderate and severe OSA, respectively. A total of 49% of the patients reported moderate or severe insomnia, and 57% experienced excessive daytime sleepiness.

**Table 1:** Characteristics of the population (*n* = 193)

Variables	Value
Gender, male, *n* (%)	131 (68)
Age (years), mean (SD, range)	59.7 (11.5, 20–84)
Education, *n* (%)	
9 years or below	47 (24)
12–13 years	87 (45)
University	59 (31)
Civil status, *n* (%)	
Married/living together	155 (80)
Living alone	38 (20)
Body composition	
BMI (kg/m^2^), mean (SD)	30.8 (4.4)
Comorbidities, *n* (%)	
Ischemic heart disease	54 (28)
Diabetes	21 (11)
Sleep-disordered breathing, mean (SD)	
Apnea-Hypopnea Index	35.6 (18.4, range 10–94)
Oxygen desaturation index	35.9 (22.1, range 10–96)
Mild OSA/moderate OSA/severe OSA, *n* (%)	21 (10)/51 (27)/121 (63)
Insomnia	
MISS score, mean (SD)	6.3 (2.2)
Daytime sleepiness	
ESS score, mean (SD)	10.8 (4.8)
ESS > 10, *n* (%)	111 (57)

### Validity and reliability testing

Based on the results from the exploratory factor analysis, three items (i.e., “I use the CPAP treatment because I want to avoid disturbing others”, “I use the CPAP because I understand that apneas are dangerous” and “I use the CPAP even if I don’t want to”) of the initial twelve were omitted. Table 2 shows the final two-factor solution containing nine items that explained 60% of the total variance. The first factor, depicting autonomous motivation, contained six items with factor loadings varying between 0.61 and 0.85. The second factor, depicting controlled motivation, contained three items with factor loadings varying between 0.79 and 0.88. The communalities for factor one and factor two varied between 0.39 to 0.73 and 0.63 to 0.78, respectively.

**Table 2:** Psychometric properties of the Motivation to Use CPAP Scale at item level (*n* = 183)

	Factor loading*	h2**	Infit MnSq***	Outfit MnSq****	Difficulty	DIF contrast across gender^a, b^	DIF contrast across sleepiness condition^a, c^	DIF contrast across insomnia condition^a, d^
Factor 1 “Autonomous motivation”/items								
1. I use the CPAP treatment because it makes me feel good.	0.721	0.554	1.10	1.07	− 0.28	0.18	− 0.06	0.13
2. I use the CPAP treatment because I want to avoid having apneas.	0.773	0.611	1.01	0.93	0.95	− 0.49	− 0.33	− 0.44
3. I use the CPAP treatment because I want to feel more alert.	0.803	0.650	0.88	0.69	0.91	− 0.44	− 0.26	− 0.26
4. I use the CPAP treatment because it feels important to me.	0.854	0.731	0.64	0.64	− 0.20	0.13	0.02	− 0.20
5. I use the CPAP treatment because my health is important to me.	0.611	0.385	1.35	1.41	0.36	0.18	− 0.37	− 0.07
6. I use the CPAP treatment because it feels good to use CPAP.	0.682	0.466	1.19	1.13	− 1.73	0.21	0.01	0.37
Factor 2 “Controlled motivation”/items								
7. I use the CPAP treatment because other people say I have to.	0.801	0.642	1.06	1.06	− 0.37	0.34	0.09	0.01
8. I use the CPAP treatment because the personnel say I have to.	0.881	0.776	0.76	0.75	− 0.10	0.01	0.27	0.06
9. I use the CPAP treatment because I have to.	0.791	0.625	1.15	1.08	0.47	− 0.36	0.11	0.01

*MnSq* mean square error, *DIF* differential item functioning

*Extraction method: Oblimin rotation with Kaiser normalization

**h2 = communalities

***Infit MnSq = information-weighted fit statistic mean square

****Outfit MnSq = outlier-sensitive fit statistic mean square

^a^DIF contrast > 0.5 indicates substantial DIF

^b^DIF contrast across gender = difficulty for males − difficulty for females

^c^DIF contrast across sleepiness = difficulty for patients with scores of the ESS > 10 − difficulty for patients with scores of the ESS ≤ 10

^d^DIF contrast across insomnia = difficulty for patients with scores of the MISS > 7 − difficulty for patients with scores of the MISS ≤ 7

The fit statistics showed that all nine items incorporated in the MUC-S had acceptable infit MnSq (0.61 to 1.35 for factor one and 0.63 to 0.78 for factor two) and outfit MnSq (0.64 to 1.41 for factor one and 0.75 to 1.08 for factor two). No differential item functioning was demonstrated for any of the items across gender, excessive daytime sleepiness or insomnia groups (Table 2). Item difficulty varied between − 1.73 and 0.95. Internal consistency was found to be high, 0.88 and 0.86. Item separation reliability and index, as well as Person separation reliability and index from Rasch, were all good (Table 3).

**Table 3:** Psychometric properties of the Motivation to Use CPAP Scale at scale level (*n* = 183)

Psychometric testing	Factor 1	Factor 2	Suggested cutoff
Standard error of measurement	1.54	1.51	The smaller the better
Ceiling effects (%)	0	5.7	< 20
Floor effects (%)	0	0.5	< 20
Internal consistency (Cronbach’s α)	0.83	0.78	> 0.7
Item separation reliability from Rasch	0.96	0.90	> 0.7
Item separation index from Rasch	4.65	3.04	> 2
Person separation reliability from Rasch	0.73	0.73	> 0.7
Person separation index from Rasch	2.30	2.63	> 2

The response frequencies for the items are shown in Table 4. There was a cumulative, but consistent response pattern, with the majority of the patients scoring strongly agree or agree, especially for the six items in factor one describing autonomous motivation. The items in factor two, describing controlled motivation, showed a more varied response pattern with fewer patients indicating high scores. No response alternative had a greater frequency of endorsement than 61%. Floor and ceiling effects were acceptable for both factors (Table 3).

**Table 4:** Descriptive statistics and item-total correlation of the Motivation to Use CPAP Scale (*n* = 183)

Factors/item	Item score distribution					Missing data (%)	Mean (SD)	Corrected item–total correlation	Skewness	Kurtosis
	Strongly agree, *n* (%)	Agree, *n* (%)	Undecided, *n* (%)	Disagree, *n* (%)	Strongly disagree, *n* (%)					
Factor 1 “Autonomous motivation”										
1. I use the CPAP treatment because it makes me feel good.	104 (58)	61 (34)	15 (8)	0 (0)	0 (0)	6.73	1.51 (0.65)	0.579	0.917	− 0.245
2. I use the CPAP treatment because I want to avoid having apneas.	132 (68)	43 (22)	4 (2)	2 (1)	0 (0)	6.22	1.31 (0.57)	0.631	2.02	4.974
3. I use the CPAP treatment because I want to feel more alert.	132 (68)	41 (21)	7 (4)	1 (1)	0 (0)	6.22	1.32 (0.57)	0.679	1.81	3.138
4. I use the CPAP treatment because it feels important to use the CPAP.	102 (53)	69 (36)	9 (5)	1 (1)	0 (0)	6.22	1.50 (0.62)	0.755	0.99–	0.666
5. I use the CPAP treatment because my health is important to me.	117 (61)	56 (29)	7 (4)	1 (1)	0 (0)	6.22	1.40 (0.59)	0.479	1.345	1.609
6. I use the CPAP treatment because it feels good to use CPAP.	68 (35)	87 (45)	21 (11)	3 (2)	2 (1)	6.22	1.81 (0.79)	0.550	1.112	2.104
Factor 2 “Controlled motivation”										
7. I use the CPAP treatment because other people say I have to.	16 (8)	41 (21)	53 (28)	32 (17)	38 (20)	6.73	3.19 (1.26)	0.580	0.003	− 1.024
8. I use the CPAP treatment because the personnel say I have to.	22 (11)	46 (24)	44 (23)	36 (19)	32 (17)	6.73	3.06 (1.29)	0.705	0.054	− 1.093
9. I use the CPAP treatment because I have to.	30 (16)	58 (30)	42 (22)	25 (14)	25 (14)	6.73	2.76 (1.28)	0.554	0.376	− 0.901

Table 5 shows the fit statistics for the latent class analysis model. The three-class model was found to be the optimal model to identify subgroups of participants (i.e., high, medium and low motivation). The Lo-Mendell-Rubin likelihood ratio test became non-significant at four classes, indicating that adding an extra class to the three-class model did not provide a better model. The largest group (*n* = 111, 61%) was called high motivation. Comparisons among patients with different motivation levels (i.e., high, moderate and low motivation) are shown in Table 6. Those with high motivation were older, had higher levels of daytime sleepiness, more problems from insomnia symptoms, a poorer global perceived health and a more positive attitude towards CPAP treatment.

**Table 5:** Latent class analysis to identify subgroups of participants (*n* = 183)

	AIC	BIC	SSABIC	Entropy	LMR test (*P* value)
2 classes	3439.79	3638.82	3445.58	0.938	396.970 (< 0.0001)
3 classes*	3392.67	3692.83	3401.40	0.944	108.462 (0.0449)
4 classes	3351.15	3752.46	3362.83	0.915	102.883 (0.775)

*AIC* Akaike’s information criterion, *BIC* Bayesian information criterion, *SSABIC* sample-size adjusted Bayesian information criterion, *LMR test* Lo-Mendell-Rubin’s likelihood ratio test

* Indicates the optimal model to identify subgroups of participants

**Table 6:** Comparisons among three subtypes of participants with different motivations classes (*n* = 183)

	High motivation (*n* = 111)	Medium motivation (*n* = 60)	Low motivation (*n* = 22)	Overall test	
				*F* test	*P* value
Age in year, mean (SE)	61.4 (1.2)^ab^	58.4 (1.4)^ac^	57.6 (2.0)	3.37	0.033
Gender (male%)*	18 (81.8)^ab^	43 (71.7)	70 (63.1)	3.51	0.061
Motivation to use CPAP, mean (SE)	21.24 (0.75)	17.66 (0.48)	16.32 (0.41)	20.37	< 0.001
Epworth Sleepiness Scale, mean (SE)	11.06 (0.63)	10.65 (0.58)	9.89 (0.71)	11.69	< 0.001
MISS score, mean (SE)	6.94 (0.41)	6.48 (0.30)	6.04 (0.22)	8.53	< 0.001
GPH score, mean (SE)	3.50 (0.08)	3.38 (0.11)	3.18 (0.23)	3.88	0.005
ACTI score, mean (SE)	8.06 (0.90)	8.68 (0.37)	9.71 (0.39)	10.69	< 0.001

*Gender variable was analysed using *χ*^2^ test

^a^Mean difference as compared with the low motivation class

^b^Mean difference as compared with the medium motivation class

^c^Mean difference as compared with the high motivation class

## Discussion

Our study using psychometric testing under both CTT and Rasch measurement theory demonstrated robust psychometric properties for the newly developed MUC-S, the first validated tool to explore how a patient with OSA perceives motivation to use CPAP treatment. We found a stable and logical two-factor solution with nine items measuring autonomous and controlled motivation explaining 60% of the total variance. Another positive aspect was Cronbach’s alpha values of 0.88 and 0.86 which suggested good reliability for the two factors. Lack of differential item functioning of items across gender, excessive daytime sleepiness or insomnia groups revealed that patients with the same latent ability had equal probability of getting an item correct. The item score distribution showed a cumulative but consistent response pattern, with the majority of the patients scoring strongly agree or agree. This was seen particularly for items in factor one describing autonomous motivation, which might be explained by data being collected before CPAP treatment was initiated. Furthermore, in the latent class analyses [36], we identified three subtypes of patients with high, medium and low motivation. Those with high motivation, the largest group (61%), had higher levels of daytime sleepiness, more problems from insomnia symptoms, a poorer global perceived health and a more positive attitude towards CPAP treatment which was deemed as logical based on data being collected before treatment initiation. However, this may change, particularly among patients experiencing side-effects [38], wherefore the trajectory of motivation, as well as factors affecting motivation at different time points is of high importance to explore in future prospective studies.

CPAP is a multifaceted treatment for a chronic disorder, and the patient’s beliefs regarding suitability of the treatment should be considered as a factor of importance for adherence [39]. Beliefs, either positive or negative, form the basis of a person’s attitude towards a phenomenon, e.g., how CPAP treatment affects health [40] which in turn can be of importance for treatment motivation. In the Motivation to Engage in Treatment (MET) theory, six cognitive and emotional internal factors predict motivation to engage in treatment: problem recognition, level of suffering, external pressure, perceived cost of treatment, perceived suitability of treatment and outcome expectancy [41]. External factors such as treatment, circumstances, situations, demographic factors and type of problems may influence the internal determinants. Understandably, clinical routines vary greatly [9], and time during patient visits is often limited [20], causing the communication focus to be more on practical aspects than behavioural aspects [16], such as motivation [12, 13], which may influence adherence to treatment negatively [6, 14]. Unlike MET, and many social-cognitive theories, which illustrate different factors that predict motivation and behaviour change, the focus of SDT is on various types of motivation and how they influence behaviour. SDT states that the type of motivation (i.e., autonomous/self-determined or controlled/non-self-determined) is more vital than the amount of motivation [21]. In a CPAP context, this means that the practitioner should strive to create a CPAP user driven by autonomous motivation. Such a patient is competent and driven by awareness of treatment benefits, in contrast to a patient who depends on controlled motivation and external regulation processes, for example, due to pressure conveyed by the CPAP practitioner. SDT incorporates a sub-theory, the Cognitive Evaluation Theory, which outlines factors that might hamper or enable different types of motivation and might form the basis of interventions to encourage more autonomous motivation. This theory suggests that more autonomous forms of motivation can be encouraged by feelings of competence, autonomy and a sense of relatedness [21]. Metacognition (i.e., defined as the ability to recognize one’s own successful cognitive processing) is linked to these feelings, and of value when trying to improve CPAP adherence, but could be difficult to measure [42]. Another aspect, important to note, is that OSA patients due to hypoxic processes might suffer cognitive impairments affecting short-term memory and concentration [43]. In a CPAP context, especially when caring for an elderly patient with severe OSA, the clinician should therefore strive to adapt the communication situation [16] to the patient’s cognitive ability [44].

Few previous CPAP studies have used interventions primarily focused on motivation, but a few have shown promising results when focusing on this aspect. Positive results have been shown using motivational enhancement therapy [12]. For example, in one study, the average nightly use of CPAP over 6 months was 99.0 min/night higher in the CPAP plus motivational enhancement therapy group, compared with the control group, an effect which was maintained over 12 months [45]. Another alternative, motivational interviewing has also proven to increase adherence [46]. Further, the relationship between the user and his/her partner might also positively influence motivation to use CPAP, since many patients tend to be “forced” into the CPAP clinic by their partner due to complaints of nightly disturbances or daytime symptoms such as fatigue and tiredness or fear of consequences [18, 19]. Future CPAP studies should explore links between CPAP adherence, autonomous motivation and the need for competence and autonomy, as well as for relatedness.

The initiation of CPAP is a complex process carried out over time. Early (i.e., 1–4 weeks) and long-term follow-up visits (i.e., 3–6 months), depending on the patients’ needs, include education on lifestyle aspects, help with practical difficulties, as well as an evaluation of treatment adherence [3]. Pathophysiology regarding OSA, technical aspects (i.e., function, care and maintenance of the device) as well as benefits and potential side-effects of CPAP, all areas adapted to the patients’ competences, should be covered [9]. Practitioners should also as previously stated consider meeting a patient with cognitive impairment [44]. Information delivered by a multidisciplinary team (e.g., the referring physician, a sleep specialist, as well as a sleep technician or CPAP nurse) is recommended [16] with the intention to reach a shared treatment decision [47]. Importantly, the increased understanding of health-behaviour change suggests the addition of specific care actions focusing behavioural change [10], as well as use of brief scales measuring attitude, motivation, habit development and shared treatment decisions in clinical CPAP care to get an understanding of important aspects. Motivation might act as a mediator or moderator in between different variables.

Future prospective longitudinal studies should explore variables and/or scales within a wide range of patient-centred areas to get an understanding of aspects unique or with synergistic impact on motivation for CPAP use. Importantly, there is no single answer to solve the complex problem of motivation/nonadherence; many factors play a role. Considering the highlighted benefits of psychosocial variables [10], the following instruments could be potential tools used together with MUC-S: ACTI [15] (i.e., measuring attitude towards CPAP), CollaboRATE and SURE (i.e., measuring aspects of shared decision making) [47] and the CPAP Habit Index-5 (i.e., measuring habit development) [48]. Another interesting scale is the Self-Efficacy Measure for Sleep Apnea [49] which explores a range of outcome expectations and aims to operationalize self-efficacy. Furthermore, it is of great importance to explore the correlation between objective CPAP use and MUC-S, and how motivation at different time-points can predict objective long-term use and healthcare utilization. The adoption of sophisticated statistical approaches (e.g., structural equational modelling) could be used to explore interactive effects of motivation between biomedical, other psychological as well as social variables on CPAP adherence.

### Strengths and limitations

This is, to our knowledge, the first study that examines motivation to use CPAP treatment in patients with OSA. No other suitable instrument for measuring motivation in this context is available. A larger sample, with data being collected at different time points, might have led to a greater variation in the response pattern. According to general recommendations for 10 observations per item, the sample size was adequate for the validation analyses of the MUC-S with its 9 items [35, 37]. A big strength of this study is the use of two important psychometric testing theories (CTT and Rasch models) [27–31]. More specifically, CTT and Rasch models provide different advantages. With the two theories, healthcare practitioners can have better understanding in the psychometric features of the MUC-S and later benefit from using the MUC-S in assessing motivation to use CPAP treatment in patients with OSA.

There are some limitations in this study. First, all data were gathered before CPAP initiation, with patients who had agreed to come to the clinic and try CPAP, which might have affected their scores. No test-retest reliability was done. Therefore, whether the MUC-S score is stable over time is uncertain. Future prospective studies are warranted to examine the stability and reproducibility of the MUC-S including patients of both genders in various age groups and with a clinically relevant range of AHI (i.e., as seen at a CPAP clinic). Second, although the sample size is decent and sufficient for the current psychometric testing, our sample size was not large enough to conduct a cross-validation. More specifically, whether the factor structure found by our exploratory factor analysis can be verified in another sample is unknown. If we attempted to do a cross-validation, the current sample size should be at least twofold to fulfil the requirement in psychometric testing (i.e., a subsample tested using exploratory factor analysis like we did and another subsample using confirmatory factor analysis with a size of 200). Third, the convenience sampling used in this study restricts the generalizability of our findings, and future large-scale multicentre studies are therefore warranted. Whether MUC-S has promising psychometric properties in other ethnicity (e.g., Asians and African Americans) is also unsure.

## Conclusion

The present study shows that the nine items included in the MUC-S were embedded in two factors measuring internal and external aspects of motivation. The scale showed good validity and reliability and operated equivalently across male and female patients. Accordingly, CPAP practitioners can use the MUC-S as a psychometrically sound tool to explore motivation related to CPAP treatment, as well as to evaluate the effects of CPAP treatment.

## Appendix

**Table. 7:** Motivation to Use CPAP Scale

Items	Response alternatives				
1. I use the CPAP treatment because it makes me feel good.	Strongly agree 5	Agree 4	Undecided 3	Disagree 2	Strongly disagree 1
2. I use the CPAP treatment because I want to avoid having apneas.	Strongly agree 5	Agree 4	Undecided 3	Disagree 2	Strongly disagree 1
3. I use the CPAP treatment because I want to feel more alert.	Strongly agree 5	Agree 4	Undecided 3	Disagree 2	Strongly disagree 1
4. I use the CPAP treatment because it feels important to use the CPAP.	Strongly agree 5	Agree 4	Undecided 3	Disagree 2	Strongly disagree 1
5. I use the CPAP treatment because my health is important to me.	Strongly agree 5	Agree 4	Undecided 3	Disagree 2	Strongly disagree 1
6. I use the CPAP treatment because it feels good to use CPAP.	Strongly agree 5	Agree 4	Undecided 3	Disagree 2	Strongly disagree 1
7. I use the CPAP treatment because other people say I have to.	Strongly agree 5	Agree 4	Undecided 3	Disagree 2	Strongly disagree 1
8. I use the CPAP treatment because the personnel say I have to.	Strongly agree 5	Agree 4	Undecided 3	Disagree 2	Strongly disagree 1
9. I use the CPAP treatment because I have to.	Strongly agree 5	Agree 4	Undecided 3	Disagree 2	Strongly disagree 1
